# Prevalence of Depression Among the Geriatric Population and Its Associated Factors: A Cross-Sectional Study in a Family Adoption Program Village in Shivamogga, India

**DOI:** 10.7759/cureus.96866

**Published:** 2025-11-14

**Authors:** Swathi H J, Anupama K, Shwetha TM, Anitha B P

**Affiliations:** 1 Community Medicine, Subbaiah Institute of Medical Sciences, Shivamogga, IND

**Keywords:** depression, elderly people, mental health services, patient health questionnaire (phq-9), prevention

## Abstract

Background: The world is aging, and the number of the aged is rising in nearly all parts of the world. There is also a demographic transition in India. Life expectancy has significantly increased in the last 70 years. Mental health, especially depression, is one of the most prevalent illnesses in the elderly. Geriatric depression, as it is also known in the older population, is linked to not only quality of life but also risk of disability, suicide, and death. This study aims to determine the prevalence of depression among the elderly population and to study the factors associated with it.

Materials and methods: It’s a cross-sectional study conducted from April 2025 to June 2025 in two centers for people in the age group above 60 years in a village with 170 participants. The study was carried out as a community-based cross-sectional study on older adults in a Family Adoption Program (FAP) village, Shivamogga, Karnataka, India. The semi-structured questionnaire was employed in the collection of data and comprised socio-demographic variables and the Patient Health Questionnaire (PHQ-9) screening scale to measure depression. IBM SPSS Statistics software, version 25.0 (IBM Corp., Armonk, NY), was used to perform statistical analysis. Descriptive statistics and chi-square tests were performed, and a p-value of <0.05 was considered statistically significant.

Results: Out of 170 participants, 60% of the participants belong to the 60-69 age group; the mean age was 66.8 years. Among them, 80% were females and 20% were males, and 87% (148) were married. The prevalence of depression was found to be 14.7%. There is a statistical association between age (<0.015) and gender (<0.017), and also the presence of diabetes (<0.004) and sleep pattern (<0.00006), and depression symptoms among elders.

Conclusion: Proper setting of geriatric mental health care programs to prevent and diagnose depression among the elderly rural population and cure and provide rehabilitation facilities to the elderly.

## Introduction

The global population is aging, with the number of elderly individuals steadily increasing across nearly all regions of the world [1]. Better healthcare, diets, and living standards have been contributing to an enormous increase in life expectancy, which has led to an aging population across the globe. According to the World Health Organization (WHO), the number of patients aged 60 years and above will increase to 2.1 billion in 2050, but in 2020, it was one billion [1].

There is also a demographic transition in India. Life expectancy has significantly increased in the last 70 years, and by 2021, around 120 million people (around 8% of the total population) were aged 60 years and older [2]. It has been estimated that this figure will increase to approximately 340 million people by 2050, which is considered one of the highest elderly populations in the country [3]. The demographic shift includes increased healthcare demand, a greater burden of chronic illness, reliance, and a decline in employment involvement.

Mental health, especially depression, is one of the most common issues in the elderly. Depression is a typical psychiatric condition that is typified by sustained melancholy, lack of interest, and disruption of normal functioning. Geriatric depression, as it is also known in the older population, is linked to not only quality of life but also risk of disability, suicide, and death [4]. In comparison with younger adults, older adults tend to have subsyndromal depression that does not fully qualify as major depressive disorder but has a severe impact on well-being [5].

Depression among elderly people is unevenly spread across the globe, depending on the region and the population group. In the last few decades, the symptoms of major depression have emerged as a significant issue for the Southeast Asian public, with the prevalence of the disorder among the elderly population estimated to be 36% [6]. Research indicates that depression among the elderly population in India has a prevalence ranging between 8.9% and a high of 62%, depending on the research methodology and criteria of depression diagnosis. [7]. Higher rates are usually reported in rural areas than in urban areas, and some studies have reported a prevalence of up to 37.8% amongst the elderly in rural areas [8]. This is highly linked with depression in late life due to the factors of chronic medical conditions, functional disability, loneliness, widowhood, finances, and the absence of a social support system.

Due to biological, psychological, and social causes, depression is more common in the elderly. The factors are age-related loss of neurotransmitters like serotonin and norepinephrine, physical disability, loss of a loved one, social isolation, retirement, and financial insecurity. Risk is further compounded by the coexistence of chronic illnesses, polypharmacy, and dependence on caregivers [9].

Since India has a rapidly growing population of aged individuals and the incidence of mental illnesses is on the rise, it is essential to diagnose and treat depression among them as soon as possible. Treating depression among the elderly will help avoid suffering, prevent catastrophic expenditure on healthcare, and enhance self-sufficiency and well-being. With this background, the current study was carried out to determine the prevalence of depression and its related variables among older adults in a Family Adoption Program (FAP) village in Shivamogga, Karnataka, India.

Despite the number of studies that have been conducted about depression among the elderly in India, these studies have been more focused on the urban areas or on small parts of the country that are located in the north and the west. The information presented in southern India, especially in Karnataka, is scanty and inaccurate, particularly in the rural region, where sociocultural and economic conditions can contribute largely to the psychological well-being of the elderly. The semi-urban district of Shivamogga, which has a large population of the elderly in rural areas, is an ideal location to evaluate these factors. The knowledge of the local prevalence and determinants of depression in older adults in this locality may be used to plan community-based mental health interventions that best meet the needs of the rural elderly demography in Karnataka.

Depression is more common among older people due to the combination of biological, psychological, and social factors. Age-related decrease in neurotransmitters such as serotonin and norepinephrine, loss of loved ones, physical disabilities, retirement, social isolation, and financial insecurity contribute significantly to its onset. The risk is further increased by the coexistence of chronic illnesses, polypharmacy, and dependence on caregivers [9].

Since India has a rapidly growing population of aged individuals and the incidence of mental illnesses is on the rise, it is essential to diagnose and treat depression among them as soon as possible. Treating depression among the elderly will help avoid suffering, save money on healthcare, and enhance self-sufficiency and well-being. With this background, the current study was carried out to determine the prevalence of depression and its related variables among older adults in an FAP village in Shivamogga, Karnataka, India.

The objectives of this study are as follows: 1. To determine the prevalence of depression among the elderly population in the community; 2. To study the factors associated with depression among elders.

## Materials and methods

Study settings

The current cross-sectional research was carried out between April 2025 and June 2025 in rural areas of Pillangiri and Hoysanahalli in Shivamogga. Both villages have a population of 3,502.

Population and sampling of the study

The study sample consisted of old people (60 years old and older) living in the targeted study areas and who met the inclusion criteria of having lived in the area for at least six months. Age verification was done via voter ID, Aadhaar card, or self-report. All the eligible participants were contacted, and those who failed to respond even after three attempts were dropped from the study. Using a 95% confidence interval (CI) and a margin of error of 5%, the required sample size was calculated based on a previous study that reported the prevalence of depression as 17%. The sample size was determined using the formula $(n = Z^{2}p(1 - p) / d^{2})$, which yielded an estimated total of 170 participants [10], where n = required sample size; Z = standard normal deviate corresponding to the desired confidence level (1.96 for 95% CI); p = estimated prevalence (17% or 0.17); and d = allowable error or precision (5% or 0.05).

All households that had elderly people were listed, and 85 participants were selected in each of the study areas using systematic random sampling, resulting in a total of 170 participants. Simple random sampling was used to identify the first house to be included in the study, and then every nth (n=5) house was picked thereafter. The study excluded participants who had medical conditions that may alter mood, like dementia, Parkinson's disease, or stroke, as well as those with severe psychiatric conditions like schizophrenia and bipolar disorder.

Study instrument

A comprehensive literature review and expert consultation were also applied in the development of a semi-structured interview schedule to be used in order to gather the perception and experience of the participants. The socio-demographic items were incorporated into the schedule, such as the education and employment history of the participants, data on contact with family members, and personal information about depression, such as substance use, the presence of chronic illnesses (such as hypertension and diabetes), and sleep habits. The individuals who had regular sleep patterns and regular sleep onset and sleep offset times were regarded as having regular sleeping patterns. Conversely, individuals with varied sleep onset and sleep offset on a daily basis were said to have abnormal sleeping habits. In the study, chronic illness was described as one that occurs for three or more months continuously or as an illness that occurs repeatedly. The moderating activity was established as a minimum of 30 minutes of activity, which was considered an adequate amount of physical activity on a daily basis. The interview schedule was pretested and then enhanced, and then administered in the local language.

Depression in the current study was screened using the Patient Health Questionnaire (nine-item version) (PHQ-9). The PHQ-9 consists of nine questions, all of which are related to a symptom of depression, and, therefore, it allows a diagnosis based on criteria. The measure of each item is a scale of zero (not at all) to three (nearly every day), with a minimum and maximum score of 0 and 27, respectively. The higher the scores, the more serious the depression was. A score of nine has been established as 88% sensitive and 80% specific to depression in elderly populations [11]. Kroenke et al. (2001) developed the PHQ-9 to measure depression in older adults. Nine items based on the Diagnostic and Statistical Manual of Mental Disorders, Fourth Edition (DSM-IV) criteria make up the PHQ-9, a standardized, self-administered depression screening tool. It has received extensive validation across a range of populations, including in India, and has shown strong criterion validity against clinical diagnosis as well as good internal consistency (Cronbach's alpha ranges from 0.78 to 0.89). The PHQ-9 is open access and freely available for use in research. Both the English and Kannada versions of the questionnaire were administered depending on the participant’s language preference [11]. Data was collected using a semi-structured questionnaire that had been pre-tested. The translated version of the questionnaire is in the form of the Kannada version (Appendix A). The questionnaire was originally written in English, which was later translated by the faculty members who were fluent in English and Kannada. Conceptual equivalence was the basis of translation as opposed to word-for-word translation because the questions were to be comprehended effortlessly by the target population. This was followed by the review of the Kannada version by a panel of faculty members of the Department of Community Medicine who were bilingual to ensure accuracy, clarity, and cultural appropriateness. An independent bilingual faculty member who had not been exposed to the original version of the questionnaire then back-translated the questionnaire into English in order to determine whether the meaning was consistent. Since the researchers created the questionnaire themselves, formal permission for translation was not needed.

Study process

The data were collected using house-to-house visits. When a participant was not available on the first visit, a follow-up visit was planned after ensuring that they could be available by contacting family members or neighbors and confirming their availability. Those subjects who were not found after three visits were treated as non-respondents. The PHQ-9 was included in the semi-structured interview schedule that was conducted according to the protocol. Any respondent with a score of nine or more on the PHQ-9 was said to be positive for depression.

Statistical analysis

The data were input to Microsoft Excel (Microsoft Corp., Redmond, WA) and were analyzed using the IBM SPSS Statistics for Windows, Version 25.0 (IBM Corp., Armonk, NY). Categorical variables were analyzed using descriptive statistics like frequency and percentages, whereas the mean and standard deviation were used to explain the continuous variables. The chi-square test was used to associate two categorical variables. A statistically significant p-value was taken to be below 0.05.

Ethical clearance

Ethical clearance was obtained from the Institutional Ethics Committee of Subbaiah Institute of Medical Sciences and Research Institute, Shivamogga (approval number IEC-SUIMS/53/2025-26). Every study participant who was enrolled in the study had the purpose of the study explained by the investigator, and informed written consent was obtained prior to inclusion.

## Results

As shown in Table 1, all 170 eligible participants were included in the analysis; no refusals were recorded. Out of 170 participants, 60% of the participants belonged to the 60-69 age group; the mean age was 66.8 years; among them, 80% were females and 20% were males; 87% (n=148) were married; 58% (98) belonged to nuclear families, while 30.5% (n=52) lived in joint families; and the majority belonged to the lower middle class. Among them, 14% (24) had a smoking history, 34% (n=58) had diabetes, and 54% (n=92) had hypertension as a comorbidity.

**Table 1:** Socio-demographic and clinical characteristics of the study participants (n = 170) Unless otherwise noted, data are expressed as frequency and percentage. Participants' average age was 66.8 years. The majority of participants were married women from the lower-middle socioeconomic class. The most prevalent comorbidities that were reported were diabetes and hypertension.

Variable	Category	Frequency (n)	Percentage (%)
Age group (years)	60–69	102	60.0
	≥70	68	40.0
Gender	Female	136	80.0
	Male	34	20.0
Marital status	Married	148	87.0
	Unmarried/Widowed	22	13.0
Type of family	Nuclear	98	57.6
	Joint	52	30.5
	Three-generation/Extended	20	11.9
Smoking history	Yes	24	14.1
	No	146	85.9
Comorbidities	Diabetes mellitus	58	34.1
	Hypertension	92	54.1
	None	20	11.8

The prevalence of depression, as presented in Table 2, was found to be 14.7% (25). In the current research study, it was noted that 45% of the respondents had no depression symptoms, and 55% of the respondents had diverse levels of depressive symptoms. One in four respondents had minimal depression (scores 1-4), and 13 had mild depression (scores 5-9). There were 9% moderate depression (scores 10-14) and only 1% moderately severe depression (scores 15-19); 5% of respondents reported severe depression symptoms (scores 20 or higher).

**Table 2:** Patient Health Questionnaire-9 (PHQ-9) data on depression symptom severity among older participants (≥60 years) The PHQ-9 has nine items with possible scores ranging from 0 (not at all) to 3 (almost every day), for a possible score of 0–27. Using common cutoffs, severity categories were established. The findings indicate that 45% of respondents did not have depression symptoms, while 55% of respondents displayed some degree of depressive symptoms. The study population's overall prevalence and range of depressive symptoms are reflected in these findings.

S. no.	Depression severity	Frequency	Percentage
1	Minimal depression (1-4)	45	27%
2	Mild depression (5-9)	22	13%
3	Moderate depression (10-14)	16	9%
4	Moderately severe depression (15-19)	1	1%
5	Severe depression (20-27)	8	5%
6	No depression (0)	77	45%

In Table 3, age and gender of the study participants were significantly related to depression symptoms in the current study. The prevalence of depression symptoms was higher in 60- to 69-year-olds (52) than in 70- to 79-year-olds (24) and over 80-year-olds (24), and statistically significant (p = 0.015). Similarly, gender also had a significant association, with females reporting a more prevalent depression (80%) than males (20%; p = 0.017). There was no statistically significant relationship between other sociodemographic characteristics, such as religion, type of family, and marital status, and depression symptoms.

**Table 3:** Association of depression status and socio-demographic characteristics This table shows the correlation between sociodemographic factors and depression in older participants, as determined by the chi-square test. The PHQ-9 score of ≥9 was used to determine depression. There were significant correlations between depression and age (p = 0.015) and gender (p = 0.017), suggesting that depression was more common in participants who were 60–69 years old and female. Marital status, family structure, and religion were among the other variables that did not reach statistical significance (p > 0.05).

Sociodemographic details		Depression present	Depression absent	P-value
Age category	60-69 years	13(52%)	90(62.0%)	0.015
	70-79 years	6(24%)	46(32.8%)	
	More than 80 years	6(24%)	9(6.4)	
Religion	Hindu	24(96%)	139(95.9%)	0.974
	Muslim	1(4%)	6(4.1%)	
Gender	Female	20(80%)	79(54.48%)	0.017
	Male	5(5%)	66(45.5%)	
Type of family	Joint family	5(20%)	46(31.7%)	0.153
	Living alone	6(24%)	16(11.03%)	
	Nuclear family	14(56.2%)	83(57.2%)	
Marital status	Married	20(80%)	128(88.3%)	0.255
	Unmarried/ divorced/widowed	5(20%)	17(11.7%)	

The association between depression symptoms and a few behavioral and health-related risk factors is compiled in Table 4. Using the chi-square test, statistical analysis was carried out. Depressive rates were significantly higher (p < 0.05) among participants with diabetes mellitus and irregular sleep patterns. Smoking, exercise, health insurance status, and hypertension did not show any statistically significant association. Clinically significant depressive symptoms were indicated by a PHQ-9 score of ≥9, which was considered depression.

**Table 4:** Association of depression status and risk factors Among the risk factors assessed, diabetes and sleep patterns were found to have a significant association with depression. Participants with irregular sleep and diabetes had a markedly higher prevalence of depression symptoms, and this association was statistically significant (p < 0.005). Other factors such as health insurance status, smoking, hypertension, and exercise did not show any significant association with depression in the study population.

Risk factors		Depression present	Depression absent	P-value
Anyhealth insurance	Do not have	23(92%)	113(77.9%)	0.104
	Have	2(8%)	32(22.06%)	
Smoking	No	22(88%)	124(85.5%)	0.742
	Yes	3(12%)	21(14.5%)	
Hypertension	No	11(44%)	67(46.2%)	0.838
	Yes	14(56%)	78(53.8%)	
Diabetes	No	9(36%)	96(66.2%)	<0.004
	Yes	16(64%)	49(33.8%)	
Sleep	Irregular	20(80%)	54(37.2%)	<0.0006
	Regular	5(20%)	91(62.8%)	
Exercise	No	23(92%)	130(89.6%)	0.718
	Yes	2(8%)	15(10.3%)	

## Discussion

A cross-sectional study on the elderly population was conducted using a community-based, cross-sectional study on 170 populations of elderly people in order to determine the prevalence of depression among the elderly population. The prevalence of geriatric depression was observed to be 14.7 in general.

Male prevalence was 20%, and female prevalence was 80%. In the present study, the prevalence rate of depression was higher among the female population. This is in line with the previous epidemiological reports that show that elderly females were more likely to be diagnosed with unipolar depression and reported more symptoms of depression than their male counterparts. In a study conducted by Vishal et al. and Rajkumar et al., they also reported that geriatric depression is common among females [12, 13]. In another research conducted by Debnath et al., the prevalence of depression among females (75%) was higher than among males (60.9%) [2]. But there are some studies in which males have a higher prevalence of depression. In a study by Vincent et al. and Nautiyal et al., the prevalence of depression was higher in males, i.e., 68.1% and 24.85%, respectively [14, 15].

In our study, the prevalence of depression was 52% in the age group of 60-69 years. In our study, depression was less prevalent in persons over the age of 80 years compared to persons who belonged to the 60-69-year cohort. Yet, research in India shows the opposite trend, with the rate of depression tending to rise with age after the age of 69 years. Studies by Swarnalatha et al. and Rajkumar et al. also reported the same result [13, 16].

Depression was more prevalent among elderly individuals who resided in a nuclear family / lived with their children in the absence of their spouse. All these indicate that geriatric individuals might be depressed because of financial dependence on others/loss of their partner. Similar outcomes were cited by Nautiyal et al., Jariwalavishal et al., and Rajkumar et al. [12, 13, 15].

The prevalence of depression in our study is 14.7%. When depression in the elderly was classified according to severity based on the scores in the study tool PHQ-9, minimal depression accounted for 26.4%, mild depression 12.9%, and moderate depression 14.7% (it includes moderate, moderately severe, and severe depression). These findings coincide with the study by Girijammal et al., which shows mild depression accounted for 15.7%, moderate depression 9.6%, moderately severe depression 10.8%, and severe depression 1.5% [17].

The meta-analysis and systematic review by Pilania et al. revealed that depression is prevalent in older people in India (34.4%: 95% CI: 29.3%-39.7) [18]. The prevalence was, however, different in the various studies, where some had a higher prevalence and others had a lower prevalence. It had also been established in earlier research that the prevalence of depression among community samples of the aged in India ranges between 13% and 43% [19]. A research study carried out in Thrissur district, Kerala, had mentioned the prevalence and severity of depression among older people (39.1%). Differences in prevalence were observed depending on the study tool employed. The Geriatric Depression Scale was applied in the studies by Singh et al., and in our case, the PHQ-9 questionnaire was used [20]. Also, a study was done in Uttarakhand regarding prevalence using PHQ-9, and they claimed a prevalence of 11.2, though a different cut-off was used in depression [21]. Another factor that could have led to the increased cases of depression among the elderly is the COVID-19 pandemic, with a number of studies indicating that there was a rise in cases of depression after the pandemic [22].

In spite of the PHQ-9 questionnaire scores not making a conclusive diagnosis of depression, this highlights the need to undertake more detailed examinations to determine the occurrence of depressive disorders, in addition to initiating therapeutic intervention. Depression among senior citizens should be identified and treated early so as to enhance the mental status and general health of the elderly citizens.

It is also more common among the elderly who have chronic illnesses. In our study, we found that the prevalence of depression among diabetes patients is 64%, and we found that there is an association between depression and diabetes. In a study by Sameera et al., the prevalence of depression among diabetics was found to be 32.5%, which was significantly higher when compared to non-diabetic patients (16.2%) [23].

Another study conducted by Trief revealed that diabetes was strongly related to depression in the geriatric population. Rajkumar et al. reported that a history of cardiac illness and diabetes exhibited statistical significance in developing depression [13, 24]. Radhakrishnan and Nayeem conducted studies that reported similar results [5].

Conducting this study in the FAP village was beneficial, as it allowed close interaction with the community and the identification of the prevalence of various health conditions, including depression among the elderly. The program thus supports health education, early detection, and planning of community-based interventions.

The present study has some limitations. It cannot show a cause-and-effect relationship between depression and other related conditions, such as diabetes or sleep disturbance, since it is cross-sectional in nature. The information obtained through self-reported measures, including sleep habits, medical history, and depressive symptoms, may be influenced by recall or reporting bias. In addition, the study sample only covered two villages of Shivamogga, thus restricting its applicability to the extensive aging population in India. Since the PHQ-9 lacks the provision of a clinical diagnosis, the application of the instrument to screen could have inflated or understated the true prevalence of depression. Moreover, it is possible that the findings were influenced by other potential confounding variables that were not well studied, including income, social support, bereavement, and cognitive decline.

## Conclusions

The present literature shows that depression is very common among the elderly. Females have a higher prevalence of depression, as do people with a history of diabetes. The findings underscore the need to diagnose and treat depression, especially in susceptible populations like those who live in urban settings and those who already have chronic conditions. Screening at the primary care level may be a good measure of depression in the elderly. The need of the hour is proper implementation of a geriatric mental health care program to prevent and diagnose depression among the elderly rural population and treat and provide rehabilitation facilities for them. By establishing a geriatric center in the rural setting, the geriatric individuals can share their thoughts and enjoy a stress-free environment.

## Appendices

Appendix A 

**Table 5:** Patient Health Questionnaire (PHQ-9) PHQ-9 score interpretation: minimal depression: 0–4; mild depression: 5–9; moderate depression: 10–14; moderately severe depression: 15–19; severe depression: 20–27.

PHQ-9				
Over the last 2 weeks, how often have you been bothered by any of the following problems? (Use “✔” to indicate your answer)	Not at all	Several days	More than half the days	Nearly every day
1. Little interest or pleasure in doing things	0	1	2	3
2. Feeling down, depressed, or hopeless	0	1	2	3
3. Trouble falling or staying asleep, or sleeping too much	0	1	2	3
4. Feeling tired or having little energy	0	1	2	3
5. Poor appetite or overeating	0	1	2	3
6. Feeling bad about yourself, or that you are a failure, o r have let yourself or your family down	0	1	2	3
7. Trouble concentrating on things, such as reading the newspaper or watching television	0	1	2	3
8. Moving or speaking so slowly that other people could have noticed? Or the opposite — being so fidgety or restless that you have been moving around a lot more than usual	0	1	2	3
9. Thoughts that you would be better off dead or of hurting yourself in some way	0	1	2	3

Kannada Translation of the PHQ-9 Questionnaire

**Table 6:** ಹಿಂದಿನ ಎರಡು ವಾರಗಳಲ್ಲಿ, ಕೆಳಗಿನ ಸಮಸ್ಯೆಗಳಲ್ಲಿ ಯಾವುದೇ ಒಂದು ನಿಮಗೆ ಎಷ್ಟು ದಿನ ಕಿರಿಕಿರಿ ಉಂಟುಮಾಡಿತು? ಮೌಲ್ಯನಿರ್ಣಯ (Scoring): ಪ್ರತಿ ಪ್ರಶ್ನೆಗೆ 0 ರಿಂದ 3 ಅಂಕ ಕೊಡಿ. 0–4 : ಖಿನ್ನತೆ ಇಲ್ಲ ಅಥವಾ ತುಂಬಾ ಕಡಿಮೆ; 5–9 : ಸಾಧಾರಣ (ಮೈಲ್ಡ್) ಖಿನ್ನತೆ; 10–14 : ಮಧ್ಯಮ (ಮೋಡರೇಟ್) ಖಿನ್ನತೆ; 15–19 : ಮಧ್ಯಮವಾಗಿ ಗಂಭೀರ ಖಿನ್ನತೆ; 20–27 : ತೀವ್ರ ಖಿನ್ನತೆ

ಕ್ರಮ ಸಂಖ್ಯೆ	ಪ್ರಶ್ನೆ	ಇಲ್ಲ (0)	ಕೆಲವು ದಿನಗಳು (1)	ಅರ್ಧಕ್ಕಿಂತ ಹೆಚ್ಚು ದಿನಗಳು (2)	ಪ್ರತಿದಿನ (3)
1	ಕೆಲಸ ಮಾಡಲು ಅಥವಾ ಯಾವದನ್ನಾದರೂ ಆನಂದಿಸಲು ಆಸಕ್ತಿ ಇಲ್ಲ				
2	ಖಿನ್ನತೆ, ನಿರಾಶೆ ಅಥವಾ ಬೇಸರದಿಂದ ನೀವು ಅನುಭವಿಸಿದ್ದೀರಾ?				
3	ನಿದ್ರೆ ಬರದೇ ಇರುವುದು, ಮಧ್ಯದಲ್ಲಿ ಏಳುವುದು ಅಥವಾ ಹೆಚ್ಚಾಗಿ ನಿದ್ರೆ ಮಾಡುವುದು				
4	ದಣಿವು ಅಥವಾ ಉತ್ಸಾಹ ಕಡಿಮೆ				
5	ಆಹಾರ ಆಸಕ್ತಿ ಕಡಿಮೆ ಅಥವಾ ಹೆಚ್ಚಾಗಿ ತಿನ್ನುವುದು				
6	ನೀವು ತಪ್ಪು ಮಾಡಿದ್ದೀರಿ, ಅಪಯಶಸ್ಸು ಎಂದು ಅನಿಸಿದ್ದು ಅಥವಾ ಕುಟುಂಬಕ್ಕೆ ಭಾರ ಎಂದು ಭಾವಿಸಿದ್ದು				
7	ಗಮನ ಕೇಂದ್ರೀಕರಿಸಲು ಕಷ್ಟ (ಉದಾ. ಪತ್ರಿಕೆ ಓದುವುದು, ಟಿವಿ ನೋಡುವುದು ಇತ್ಯಾದಿ)				
8	ನಿಮ್ಮ ಚಲನೆ ಅಥವಾ ಮಾತು ಸಾಮಾನ್ಯಕ್ಕಿಂತ ನೀವಾಗಿ ಇದ್ದಿತ್ತೆ? ಅಥವಾ ಬಹಳ ಆತುರದಿಂದ ಚಲಿಸಿದ್ದೀರಾ?				
9	ನೀವು ಸಾಯುವುದು ಅಥವಾ ನಿಮ್ಮನ್ನು ನೀವೇ ಹಾನಿ ಮಾಡಿಕೊಳ್ಳುವುದು ಒಳ್ಳೆಯದು ಎಂದು ಯೋಚಿಸಿದ್ದೀರಾ?				
